# Time to integrate spiritual needs in health care

**DOI:** 10.1016/j.lanepe.2023.100648

**Published:** 2023-05-02

**Authors:** The Lancet Regional Health – Europe

Over the past decades there has been a growth in the field of spirituality and health research showing a positive influence of spirituality and spiritual care, on both mental and physical health outcomes. Providing spiritual care to patients with serious illness has been associated with better end-of-life outcomes, and unmet spiritual needs can be associated with poorer patient quality of life and wellbeing. As per literature estimates, spirituality is important to most patients with serious illness (71–99%) and spiritual care is frequently desired by patients with serious illness (50–96%). Despite these findings, spiritual needs of patients with serious illness are frequently unaddressed within medical care—estimates of patients not receiving spiritual care range from 49 to 91%.

One of the root causes for not accounting for spirituality in medical care is the lack of consensus on the understanding of what spirituality is, and how prevalent spiritual needs are. Spirituality is a broad and complex concept, with no single consensus definition in medical practice and is often considered taboo. According to the International Consensus Conference on Spiritual Care in Health Care, spirituality is the way individuals seek ultimate meaning, purpose, connection, value, or transcendence. Spirituality can include organised religion but extends beyond to include ways of finding ultimate meaning by connecting, for example, to family, community, nature, or things people hold sacred.

Although research supports the importance of spirituality for health, in supposedly secular societies, spiritual matters are rarely addressed in health care and are not considered as a critical pillar of holistic health care. This lack of attention to spiritual needs is partly due to limited information on whether adults in secular cultures have spiritual needs and, if so, what kinds of spiritual needs. To understand the prevalence of spiritual needs, a new Article published in this issue of *The Lancet Regional Health – Europe*, examined the spiritual needs in the Danish population. This is the first and largest study on spiritual needs to date that quantitatively demonstrates that spiritual needs are common among Danes. Of included participants, 82% reported at least one strong or very strong spiritual need (among one of the four: religious, existential, generativity, and inner peace) in the past month. The participants scored highest on inner peace needs, followed by generativity, then existential, and lastly, religious needs.

These findings have important implications for public health policies and clinical care as it testifies that spiritual care is widely wished for, which might be uncomfortable for secular health planners to admit. Although this study is from Denmark, the outcomes might be similar in other post-secular European cultures. In the linked Comment to this Article, Andrew Tomkins makes a strong impactful argument that “this paper is a challenge to those who assume that ‘faith is dead’ in our modern world. It has important implications for all governments and healthcare workers with responsibility for planning health services for those facing serious or terminal illness …. But whose interests are more important - our patients' welfare or our own preconceptions?”

How to approach spiritual needs and give spiritual care in a pragmatic way can be challenging in health care. In their Correspondence, Richard Armitage argues that spiritual care should extend to include all patients that desire it, not merely those facing serious or terminal illness. However, the increasing demands on primary health care, the underinvestment in general practice, and the consequential dearth of workforce morale in many countries, pose substantial barriers to the inclusion of such vital care into standard practice within primary health care.

These concerns are valid, but the needs of the patients should be given paramount importance for their health and wellbeing. Incorporating small changes can make a big difference in the long run. There is a need for increasing awareness among clinicians and health professionals about the benefits of providing spiritual care. Examples include training for clinicians aimed at enhancing their spiritual care competencies; or taking a spiritual history, with simple questions or with more advanced tools, to allow clinicians to understand patients more fully. This approach can then lead to referrals to a spiritual care specialist, if needed. It is also important to understand that spiritual care can be provided despite the provider's personal beliefs (religious, spiritual, atheist, etc) and whether the provider shares the same beliefs as their patient or not.

More research on clinical effectiveness of spiritual care interventions is needed and large randomised controlled trials are required to examine the efficacy of spiritual care interventions on clinical outcomes. As with all disciplines, more granular understanding of spiritual needs and their outcomes is needed but care for the spiritual dimension of health is warranted along with the physical, mental, and social aspects that are included under the core umbrella of the health-care system.

